# Special Issue “Gene Doping Control”

**DOI:** 10.3390/ijms27062631

**Published:** 2026-03-13

**Authors:** Teruaki Tozaki

**Affiliations:** Genetic Analysis Department, Laboratory of Racing Chemistry, Utsunomiya 320-0851, Japan; ttozaki@lrc.or.jp

## 1. Introduction

Horse racing is a unique sporting domain in which competitive fairness and pedigree authenticity are linked institutionally and inseparably. In Thoroughbred racing, “clean sport” encompasses not only fair competition but also the accuracy of studbook registration, the maintenance of genetic diversity, and considerations of animal welfare. Therefore, the integrity of the sport depends on ensuring that horses compete without the artificial manipulation of performance or genetic identity. This fundamental principle is shared with equestrian sports and, more broadly, human sports, where doping has long been prohibited to preserve public trust and sporting legitimacy.

To uphold these principles, international governing bodies such as the International Federation of Horseracing Authorities (IFHA), the Fédération Equestre Internationale (FEI), and the World Anti-Doping Agency (WADA) have developed rules, standards, and guidelines tailored to their respective sporting domains [1,2]. In horse racing, anti-doping systems grounded in analytical science have been established at an early stage. Historically, these systems have focused on detecting prohibited substances and ensuring regulatory compliance.

Traditional anti-doping efforts have primarily targeted pharmacological doping, including the use of low-molecular-weight compounds, peptides, and protein-based agents. Advances in analytical chemistry, especially mass spectrometry, have enabled the highly sensitive and reproducible detection of these substances and have formed the backbone of modern anti-doping programs [3,4]. This domain represents a mature scientific field with well-established methodologies.

However, rapid advances in medical science and molecular biology have fundamentally changed the landscape of doping practices. The emergence of gene doping represents a paradigm shift that challenges the conceptual and technical foundations of conventional anti-doping strategies [5,6]. Gene doping includes the introduction of exogenous genetic material (transgenes), application of genome editing technologies, and misuse of nucleic acid–based therapeutics such as oligonucleotides. These interventions differ qualitatively from classical pharmacological doping and require fundamentally different detection strategies and scientific evaluation frameworks.

In this Editorial, we argue that these emerging challenges cannot be adequately addressed within conventional, testing-centered anti-doping frameworks and instead require the recognition of anti-doping as an integrated scientific discipline, herein referred to as “anti-doping science,” with horse racing serving as a central model.

## 2. Conventional Doping and Gene Doping

The essential differences between conventional and gene doping are summarized in Table 1, highlighting the fundamental shift from transient biochemical manipulation to direct and potentially irreversible intervention in genetic information with profound regulatory and ethical implications. Unlike pharmacological agents, which act at the metabolic or physiological levels and are transient in nature, gene doping targets genetic and genomic structures and may result in long-lasting or permanent alterations. Consequently, molecular biology approaches, such as polymerase chain reaction-based assays and next-generation sequencing, have become indispensable tools for gene doping control [7].

In the context of horseracing, the implications of gene doping extend far beyond the unfair enhancement of athletic performance. Of particular concern is the issue of gene-edited animals, which directly threatens the integrity of the Thoroughbred studbook system. If intentionally gene-edited horses enter racing or breeding populations, the consequences would include erosion of studbook credibility, disruption of genetic integrity within breeding populations, and loss of public confidence in the racing industry. Thus, horse racing must confront gene doping not only as a matter of competitive fairness but also as an existential challenge to its institutional foundations. Although genome editing technologies may have legitimate therapeutic potential in veterinary medicine, the boundary between medical treatment and performance enhancement remains scientifically and ethically ambiguous. This ambiguity reinforces the necessity for a robust interpretative framework rather than binary regulatory classifications.

Detection of gene doping and editing relies on molecular techniques such as polymerase chain reaction and next-generation sequencing, which enable the identification of foreign genetic elements, edited genomic regions, or abnormal genetic signatures inconsistent with naturally occurring variations [8]. These methods require robust reference databases, high-confidence variant interpretations, and the careful discrimination between artificial modifications and natural genetic diversity [9]. Importantly, because these approaches interrogate genetic information, they introduce ethical and legal considerations that fundamentally differ from those associated with conventional drug testing [10].

As gene doping control advances, the importance of interpretation increases. Analytical data must be evaluated considering technical artifacts, biological variability, and multiple possible intervention scenarios. Thus, the scientific assessment of causality and intent becomes as important as the detection itself, marking a shift from compliance-driven, substance-oriented testing to a comprehensive scientific discipline centered on interpretation, causality, and uncertainty.

The relevance of this discussion extends beyond horse racing. Knowledge and methodologies developed in the racing domain provide important insights into human sports, where gene doping control is still largely conceptual. A comparison of the gene doping–related challenges in horse racing and human sports is presented in Table 2. While both domains share concerns about fairness, the ethical implications of genetic testing are particularly pronounced in human sports, where genomic analyses may reveal information about athletes’ family members.

In human sports, the direct application of gene doping control strategies developed for horse racing is neither straightforward nor guaranteed. Therefore, the question of how far sports authorities may go in requiring genetic analyses when gene doping is suspected remains unresolved. Establishing appropriate boundaries and safeguards is essential before testing becomes routine. These issues must be proactively addressed as technological capabilities continue to advance rapidly.

## 3. Anti-Doping Science for Gene Doping

Against this background, this Editorial proposes that the challenges described above should be reframed within the unified conceptual framework of anti-doping science. The studies assembled in this Special Issue collectively illustrate the methodological diversification required for contemporary gene doping control. Direct molecular strategies now enable the identification of transgenes, edited loci, and candidate gene-doping constructs across multiple biological matrices, including conventional blood samples and dried blood spots. Analytical advances further extend detection capacity to therapeutic nucleotides through sequence-independent high-resolution mass spectrometry approaches Complementing these strategies, investigations of circulating microRNAs exemplify indirect detection paradigms that interrogate biological responses rather than exogenous genetic material itself. Taken together, these contributions demonstrate that gene doping represents a heterogeneous spectrum of interventions—spanning transgenic DNA, genome editing, and regulatory RNA modulation—that necessitates integrative and interpretative scientific frameworks and collectively advance the methodological foundation for future anti-doping testing strategies.

Rather than viewing anti-doping solely as a collection of testing procedures or regulatory obligations, it should be recognized as a scientific discipline grounded in hypothesis-driven evaluation of biological deviations arising from intentional human intervention. Anti-doping science integrates analytical chemistry, molecular biology, genomics, veterinary and human medicine, forensic science, statistics, ethics, and legal scholarship. Its objective is not merely detection, but rigorous interpretation within scientifically and ethically robust boundaries.

The disciplinary foundations of anti-doping science are summarized in Table 3. By explicitly defining this field, anti-doping efforts can advance beyond routine testing toward systematic methodological innovation and structured evaluation of uncertainty.

As illustrated by gene doping and gene-edited animals, future anti-doping science will inevitably engage with highly sensitive genetic information. Therefore, designing governance models that balance sporting fairness with the protection of individual and familial genetic privacy should be recognized as a core research objective. Horse racing, with its long-standing integration of pedigree verification, genetic testing, and anti-doping control, is uniquely positioned as a model system for this emerging field.

Ultimately, the definition and promotion of anti-doping science is not merely an investment in its enforcement. It is an investment in the credibility and sustainability of sports. By grounding anti-doping efforts in rigorous science and ethical reflection, sporting communities can respond constructively to technological innovation while maintaining public trust. In an era of rapid progress in molecular biology and medicine, anti-doping science offers a coherent framework for safeguarding the integrity and long-term sustainability of horse racing, equestrian sport, and human sport.

## 4. Conclusions

In this Editorial, we argue for a shift in how anti-doping should be understood and practiced, beyond compliance-driven testing alone. This field integrates analytical chemistry, molecular biology, genomics, bioinformatics, ethics, and legal science to address intentional human intervention in biological systems that undermine sports integrity. Using Thoroughbred racing as a central model, we further argue that equine sports provide a uniquely advanced framework for anticipating future challenges in human sports, particularly in the detection of gene doping and genome editing. In this context, gene-edited animals represent not only a novel form of enhancement but also a systemic risk to the structural foundations and societal credibility of sports, requiring anti-doping strategies that extend beyond post-competition testing. Moreover, as genomic technologies have become essential tools for gene doping control, ethical considerations and the protection of genetic information must be regarded as intrinsic scientific constraints rather than external regulatory issues. By explicitly defining anti-doping science and clarifying its scope, methodological boundaries, and ethical foundations, this Editorial aims to provide a unifying framework that supports future research, policy development, and international harmonization in gene doping control, thereby contributing to the long-term sustainability of clean racing, equestrian sports, and human sports.

## Figures and Tables

**Table 1:** Comparison between conventional and gene doping.

Category	Conventional Doping	Gene Doping
Targets	Low-molecular-weight compounds, peptides, proteins	Transgenes, genome-edited loci, therapeutic nucleotides
Biological level	Metabolic/physiological modulation	Genetic and genomic modification
Detection methods	Mass spectrometry	PCR, qPCR, dPCR, NGS
Detectability	Limited by pharmacokinetics	Potentially long-lasting or permanent
Risk of false interpretation	Contamination, metabolic variability	Mosaicism, natural genetic variation, technical artifacts
Ethical considerations	Fairness and athlete/animal health	Fairness, genetic privacy, familial implications

**Table 2:** Gene doping control in horse racing and human sports.

Aspect	Horse Racing	Human Sports
Governing bodies	IFHA, national racing authorities	WADA, international federations
Key concern	Competitive fairness and pedigree authenticity	Competitive fairness and individual integrity
Central genetic issue	Gene-edited animals affecting studbooks	Genome editing affecting athletes and families
Impact beyond competition	Breeding population, genetic diversity	Family genetic privacy
Ethical focus	Genetic integrity of animals and industry trust	Consent, privacy, data protection

**Table 3:** Disciplines contributing to anti-doping science.

Discipline	Contribution to Anti-Doping Science
Analytical chemistry	Detection of prohibited substances
Molecular biology	Detection of gene doping and genome editing
Genomics and bioinformatics	Reference databases and variant interpretation
Forensic science	Scenario analysis and causality assessment
Statistics	Quantification of analytical uncertainty
Ethics	Protection of genetic privacy and rights
Law/governance	Regulatory frameworks and compliance
